# Improving healthcare quality in Sudan: situation and factors influencing healthcare professionals’ engagement

**DOI:** 10.1186/s12913-025-13481-3

**Published:** 2025-09-30

**Authors:** Swsan A. M. Elsharif, Elaf M. H. Abdelraheem, Hajar Saad Salih, Arwa Nasr, Mohamed Eltayeb Elnour, Reem A. A. Mohamedelmugadam, Zuhal Yahya Mohammed Omer, Reem Azmi A. Yousif, Ayat Abdelbagi Ahmed Mohamed

**Affiliations:** 1 Faculty of Medicine, University of Khartoum, Khartoum, Sudan; 2 Faculty of Medicine, University of Bahri, Khartoum, Sudan; 3 Faculty of Nursing, Alzaim Alazhari University, Khartoum, Sudan

**Keywords:** Quality improvement, Health systems, Healthcare professionals, Quality of care, Leadership

## Abstract

**Background:**

Quality improvement (QI) projects depend on the active involvement of healthcare professionals. However, their engagement remains suboptimal, specially in humanitarian settings such as Sudan. Our study aimed to describe healthcare professionals’ engagement and to identify facilitators and barriers to conducting QI projects.

**Methods:**

An online-based cross-sectional survey was conducted in Sudan between July and November 2024 using convenience sampling. The survey was distributed to healthcare professionals through different social media platforms. Data were manually cleaned in Excel sheet and analysed using Statistical Package for the Social Sciences Version 20 (SPSS 20). Chi Square test, Mann Whitney U test and Kruskal Walis test were used to identify factors associated with experience and self-efficacy in QI.

**Results:**

A total of 1007 healthcare professionals were included in the study; the mean age was 27 ± 5 years, and the majority (67.9%) were females. Most of the participants (74.7%) were physicians, and (15.7%) were nurses. Only (18%) of participants reported that they have prior experience with QI projects. Older age, male gender and increased years of experience were found to be significantly associated with QI experience (p value < 0.05). Factors that influence self-efficacy in conducting QI projects were older age groups and increased years of experience, in addition to professional development opportunities such as formal training in QI, professional workshops in QI, and QI organisational membership. Barriers to conducting QI projects were lack of organisational support (59.1%), no access to QI content (48.6%), lack of time (39.8%), and lack of mentorship (31.5%).

**Conclusion:**

The study reveals low engagement of healthcare professionals in QI projects. Organisational support and professional development opportunities are essential to ensure effective healthcare professionals’ engagement in QI projects, thereby enhancing the quality of care and ensuring favourable outcomes.

**Supplementary Information:**

The online version contains supplementary material available at 10.1186/s12913-025-13481-3.

## Introduction

The framework of the World Health Organisation (WHO) 2007 determines the importance of quality improvement (QI) in strengthening the healthcare systems in developing countries and improving patient outcomes [1]. Despite the worldwide approaches to enhance QI initiatives, their effectiveness and application in developing countries are still suboptimal [2].

Experts define QI as continuous performance enhancements and a set of technical methods [2]. Key observations of experts are that QI has major effects in poor-resource environments. The knowledge about QI is not spread enough in developing countries and needs continuous efforts to be achieved. Also, we need to integrate QI in health systems within the WHO Six Building Blocks, which include: providing healthcare services, healthcare worker enhancement, health system financing, information systems, medical products, and leadership and governance [2].

Quality improvement is challenging in settings where healthcare providers work with maximum effort in poor-resource environments, i.e. in low-income countries, it is difficult to provide resources to support quality improvement [3]. Integrating quality improvement principles and policies that increase the efficiency of the healthcare system is more urgent, as patients in these settings gain more access to care [4].

The success of quality improvement initiatives depends mainly upon healthcare professionals’ (HCPs) engagement; their active involvement is essential, as they provide valuable insights and expertise [5, 6]. Many studies suggested that active involvement of healthcare professionals in QI Projects results in superior outcomes than those with minimal involvement [7, 8]. However, despite the growing evidence of HCPs’ role in the success of QI projects, their engagement is considered low [9].

While factors that influence HCPs’ engagement may differ across different contexts, common factors were identified. These factors can be grouped into three levels: organisational level, HCP level, and the QI project level. At the organisational level, culture and climate, human resources, infrastructure, and institutional priorities were the identified factors. At the HCPs’ level, factors related to their motivation, organisational role and collaboration with groups were recognised. At the QI project level, the approach to QI, knowledge and training in QI, and access to QI mentors were found to influence HCPs’ engagement [10].

In Sudan, the ongoing conflict has led to a huge humanitarian crisis, which has caused more fragility in the healthcare system. The war has led to wide displacement, with approximately 11 million people internally displaced and 3.1 million refugees in neighbouring countries [11]. This wide displacement has placed unprecedented pressure on healthcare services. Approximately 60–70% of health facilities are disrupted, which makes access to primary healthcare more difficult [12]. Displacement is one of the main factors that maximise healthcare fragility in Sudan. The influx of displaced individuals into host communities, where healthcare resources are already poor, has led to a huge humanitarian crisis. A critical intersection between people in need and humanitarian needs is evident in many areas in Sudan [13].

In this context, QI knowledge has become extremely essential among healthcare providers. QI initiatives need to focus on enhancing healthcare outcomes, particularly in poor-resource settings [2]. By implementing effective QI strategies, healthcare professionals can better deal with challenges such as shortages of medical supplies, healthcare workforce gaps, and inadequate infrastructure [14]. This makes QI a crucial tool for decreasing the impact of war and displacement on Sudan’s fragile health system. Thus, our study aims to assess HCPs’ engagement in QI projects and the factors that influence their engagement to better understand the situation and to inform evidence-based policymaking.

## Methods

### Study design and settings

This is a descriptive cross-sectional online-based national study conducted in the 18 states of the Republic of Sudan from July to November 2024. The study reporting followed the STrengthening the Reporting of OBservational studies in Epidemiology (STROBE) guidelines [15].

### Study participants

The study focused on the frontline healthcare professionals: Doctors/Physicians, Nurses, Lab scientists, Dentists, Physical Therapists, and pharmacists who worked in governmental and private healthcare centres in Sudan.

### Sample size and sampling techniques

A convenience sampling method was used to recruit the study participants. Sample size was calculated using the Fischer et al. formula: n = z^2^P (1-P)/d^2^. Where 50% response distribution was applied due to the absence of previous data, with a 95% confidence level. The minimum sample size was calculated to be 386. A design effect was applied in this study, doubling the sample size to 772 participants, to minimise the limitations of convenience sampling. We collected 1007 responses during the study period to increase statistical power and account for potential exclusions due to incomplete responses [16, 17].

### Data collection tool

The questionnaire used in our study was adapted from an instrument used in a similar study [18]. The original instrument was modified to align with the local context and was tested on 50 participants before data collection to ensure clarity and comprehensiveness. The final version was reviewed by the researchers and revised by a group of QI experts before administering it to the study participants.

### Data collection methods

The anonymous questionnaire was administered in English using Google Forms. It was distributed on various social media platforms, including LinkedIn, WhatsApp, Telegram, Facebook and X. Only one response was recorded per participant in this study to ensure data integrity.

### Study variables

- Independent variable.

Age, Gender, State/ Country of origin, Professional background, Years of clinical practice,

- Dependent variables.

Experience and self-efficacy in conducting quality improvement activities.

### Data management and analysis

Data was exported to Excel 2022, cleaned and revised variable by variable, then coded and imported to Statistical Package for the Social Sciences version 20 (SPSS 20). Chi-square test was used to test the association between experience in QI and demographic characteristics. Mann-Whitney U test and Kruskal-Wallis test were used to identify factors associated with self-efficacy in QI.

### Ethical considerations

Ethical approval was obtained from the White Nile State Ministry of Health Review Board under the approval number **0230111**. Informed consent was obtained from all participants, clearly explaining the research purpose and their right to withdraw from the study at any time. The study adhered to the ethical standards outlined in the Declaration of Helsinki, ensuring the privacy and protection of all participants. Data were anonymised and handled confidentially by the research authors.

## Results

### Healthcare professionals characteristics

A total of 1007 healthcare professionals were included in the study; the mean age was 27 ± 5 years, and the majority were females, 684 (67.9%). Three-quarters, 722 (76.7%) of the participants worked in urban settings, and more than half, 533 (52.9%) worked in a state government hospital. About one-third of the participants, 337 (33.5%) were from Khartoum, and only 40 (4%) were from the West. Most of the participants, 752 (74.7%) were physicians, followed by nurses 152 (15.7%), pharmacists 42 (4.2%), dentists, lab scientists and physical therapists, 55 (5.5%). About 693 (68.8%) of the participants had worked as healthcare professionals for less than two years (Table 1).

**Table 1:** Healthcare professionals’ characteristics and their association with quality improvement experience

Variable	Total *N*		Experience in QI				
			No		Yes		*P* value
**Age in years**							**0.000**
18–33	904	(89.8%)	761	(84.2%)	143	(15.8%)	
34–49	96	(9.50%)	61	(63.5%)	35	(36.5%)	
50 and more	7	(0.70%)	4	(57.1%)	3	(42.9%)	
**Gender**							**0.001**
Male	323	(32.1%)	245	(75.9%)	78	(24.1%)	
Female	684	(67.9%)	581	(84.9%)	103	(15.1%)	
**Professional location**							0.293
Urban	772	(76.7%)	631	(81.7%)	141	(18.3%)	
Rural	149	(14.8%)	128	(85.9%)	21	(14.1%)	
Suburb	86	(8.50%)	67	(77.9%)	19	(22.1%)	
**Employment setting**							0.222
State government hospital	533	(52.9%)	434	(81.4%)	99	(18.6%)	
Private hospital	163	(16.2%)	127	(77.9%)	36	(22.1%)	
University health center	207	(20.6%)	178	(86.0%)	29	(14.0%)	
Community health center	104	(10.3%)	87	(83.7%)	17	(16.3%)	
**Years of experience**							**0.000**
Less than 2 years	693	(68.8%)	600	(86.6%)	93	(13.4%)	
2–6 Years	216	(21.4%)	162	(75.0%)	54	(25.0%)	
More than 6 years	98	(9.70%)	64	(63.3%)	34	(34.7%)	
**Clinical training**							0.069
Physician	752	(74.7%)	607	(80.7%)	145	(19.3%)	
Nurse	158	(15.7%)	136	(86.1%)	22	(13.9%)	
Pharmacist	42	(4.20%)	37	(88.1%)	5	(11.9%)	
Lab scientist	17	(1.70%)	17	(100%)	0	(0.00%)	
Dentist	33	(3.30%)	26	(78.8%)	7	(21.2%)	
Physical therapist	5	(0.50%)	3	(60.0%)	2	(40.0%)	
**Region**							0.187
Khartoum	337	(33.5%)	274	(81.3%)	63	(18.7%)	
North Sudan	264	(26.2%)	206	(78.0%)	58	(22.0%)	
Central Sudan	191	(19.0%)	164	(85.9%)	27	(14.1%)	
West Sudan	40	(4.00%)	35	(87.5%)	5	(12.5%)	
East Sudan	175	(17.4%)	147	(84.0%)	28	(16.0%)	

P-value: significance level at 95% confidence interval

**Fig. 1:**
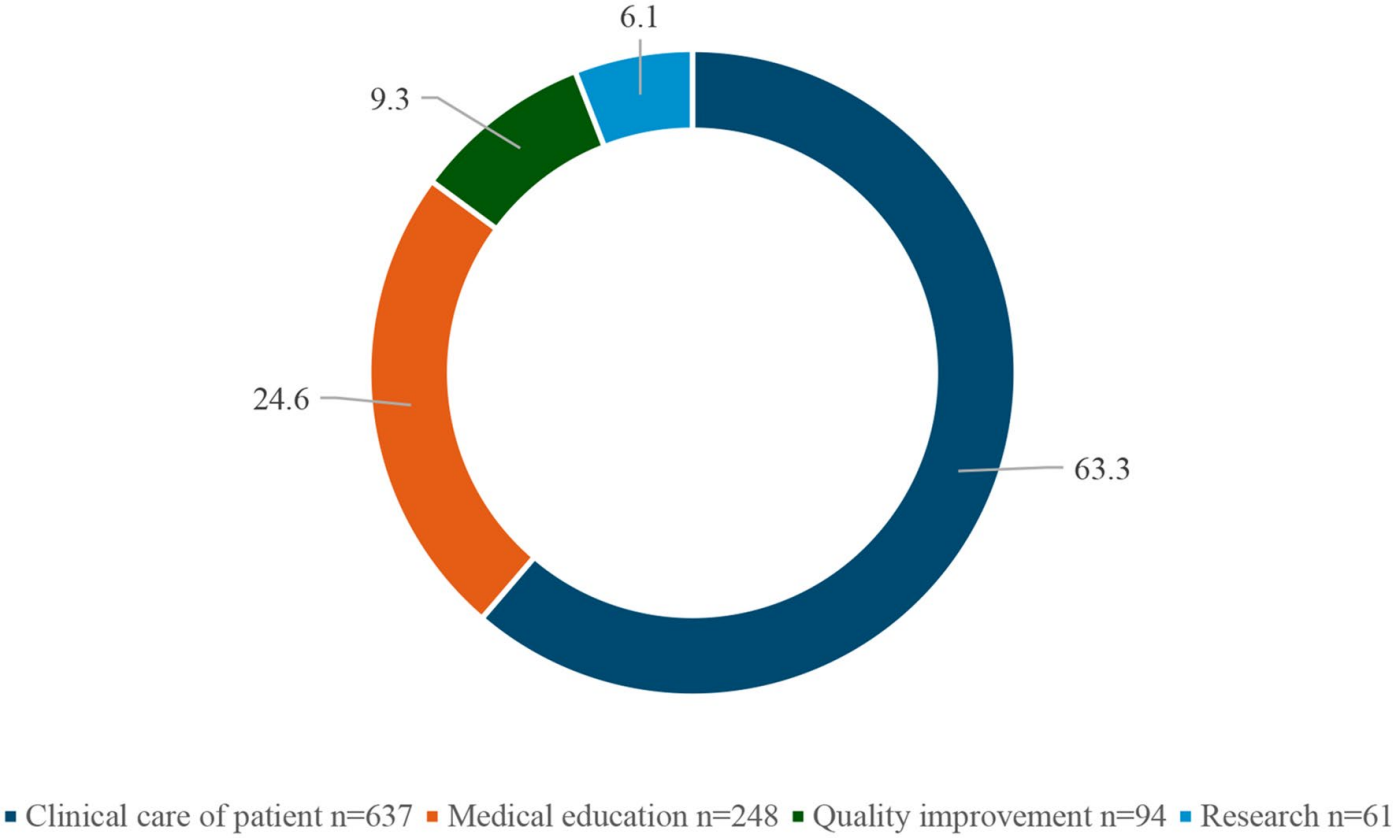
Frequency of healthcare professionals’ engagement in professional activities during a typical workweek

During a typical workweek, 637 (63.3%) of the participants spent most of their time in the clinical care of patients, and only 94 (9.3%) and 61 (6.1%) in QI and research, respectively (Fig. 1). Interestingly, only 181 (18%) of participants reported that they have prior experience with quality improvement projects.

Age was significantly associated with experience in QI (P value < 0.001). The age range of 18–33 years (15.8%) was more likely to have no experience with QI than the age range of 34–49 years (36.5%) and 50 or more years (42.9%). Gender was also significantly associated with expertise in QI (P value = 0.001), as males reported more experience in QI (24.1%) than females (15.1%). There was no significant association between clinical training, professional location and employment settings with expertise in QI (P value > 0.05). However, there was a significant difference in QI experience (P value < 0.001), between the years of experience. The percentage of healthcare professionals with more than 6 years of experience, and with 2–6 years’ experience, had reported having significantly more QI experience than healthcare professionals with less than 2 years of experience (Table 1**).**

### Healthcare professionals’ engagement in QI projects

Of the 181 healthcare professionals who had QI experience, 143 (79%) had participated in a QI project, 35 (19.3%) had advised or coached, and 35 (19.3%) had led a QI project or initiative. Only 22 (12.2%) of healthcare professionals held a formal administrative title related to QI. Healthcare professionals had been involved in QI activities for an average of 1 (IQR 1–2) year and reported being involved in 2 (IQR 1–3) QI Projects. More than one-third 71 (39.2%) reported receiving a course in QI before graduation, and 66 (36.5%) reported that the course was part of their university curriculum.

Professional development opportunities that healthcare professionals utilized were formal QI training in their hospital 21 (11.6%), formal QI training outside their hospital 12 (6.6%), professional workshops or seminars in QI 32 (17.7%), online courses in QI 21 (11.6%), QI conference 12 (6.6%), QI membership 9 (5%), mentor in QI 25 (13.8%), and peer networking opportunities in QI 31 (17.1%). About 83 (45.9%) of healthcare professionals reported that their hospital provided them with dedicated time to participate in QI, and 78 (43.1%) reported that their hospital recognised and rewarded their participation.

Healthcare professionals stated that the most successful QI project that they were involved in resulted in improved patient safety or reduction in medical errors 133 (73.5%). Other reported outcomes were: implemented clinical guidelines 76 (42%), improved patient/family centeredness 31 (17.1%), reducing waits and delays in care 45 (24.9%), increasing efficiency of clinical care 75 (41.4%), and improving equity 50 (27.6%).

### Factors and barriers affecting QI engagement

The factors that were attributed to the success of QI projects were formal training in QI methods 77 (42.5%), experience with QI 66 (36.5%), mentorship in QI 55 (30.4%), hospital rewards participation in QI projects 38 (21%), connection to a network of QI professionals (21.5%), and interprofessional QI teams 21 (11.6%).

Personal strengths that contributed to the effectiveness of healthcare professionals in QI were the ability to identify problems that need to be fixed 131 (72.4%), reflecting on and learning from experiences 98 (54.1%), curiosity 56 (30.9%), facilitating ways to enable people to share ideas 30 (16.6%), and being a team player 21 (11.6%).

**Fig. 2:**
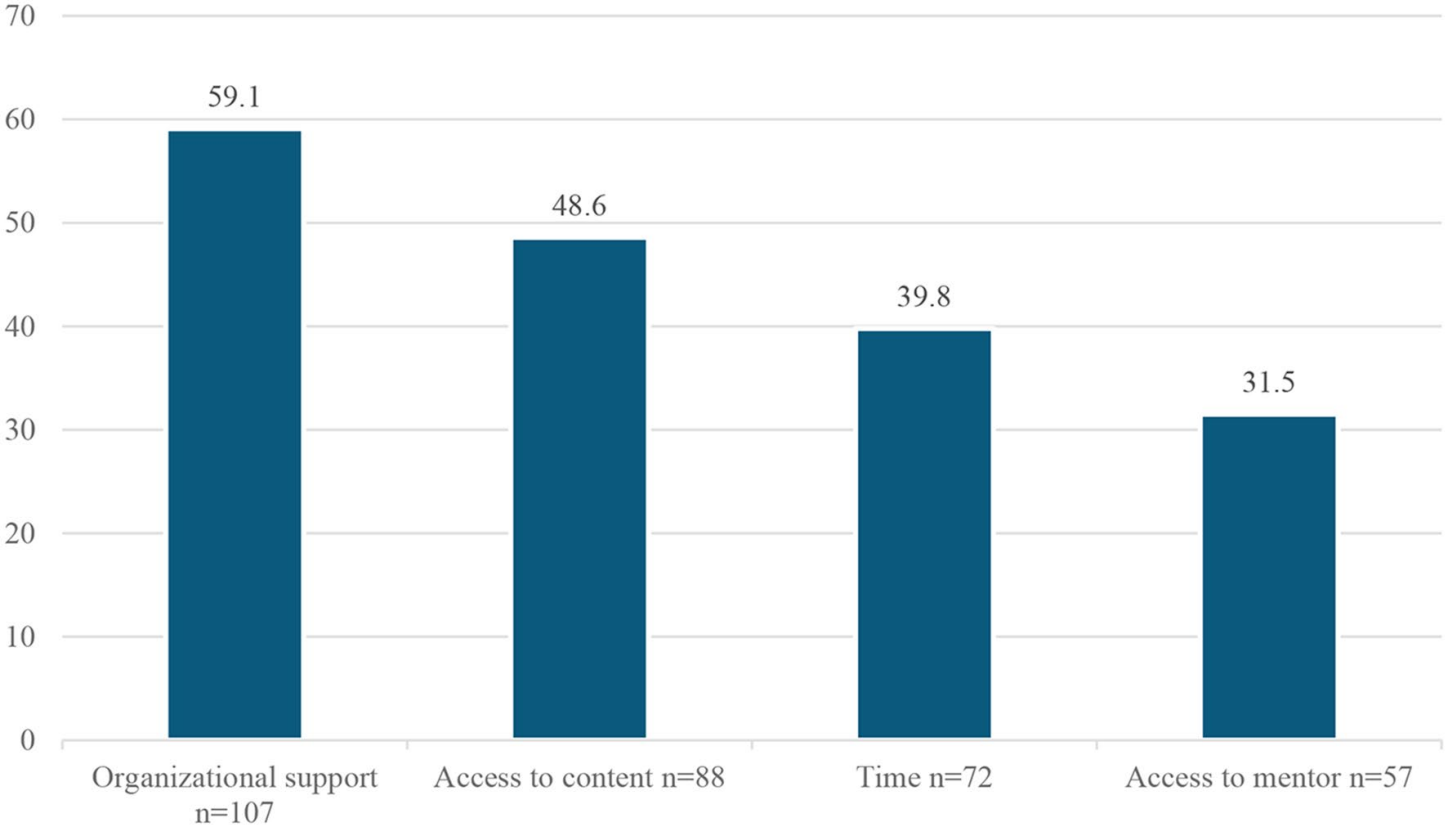
Barriers to conducting QI projects as reported by healthcare professionals

Healthcare professionals reported the most frequent barriers to conducting QI projects were lack of organizational support 107 (59.1%), no access to QI content 88 (48.6%), lack of time 72 (39.8%), and lack of mentorship 57 (31.5%) (Fig. 2).

**Fig. 3:**
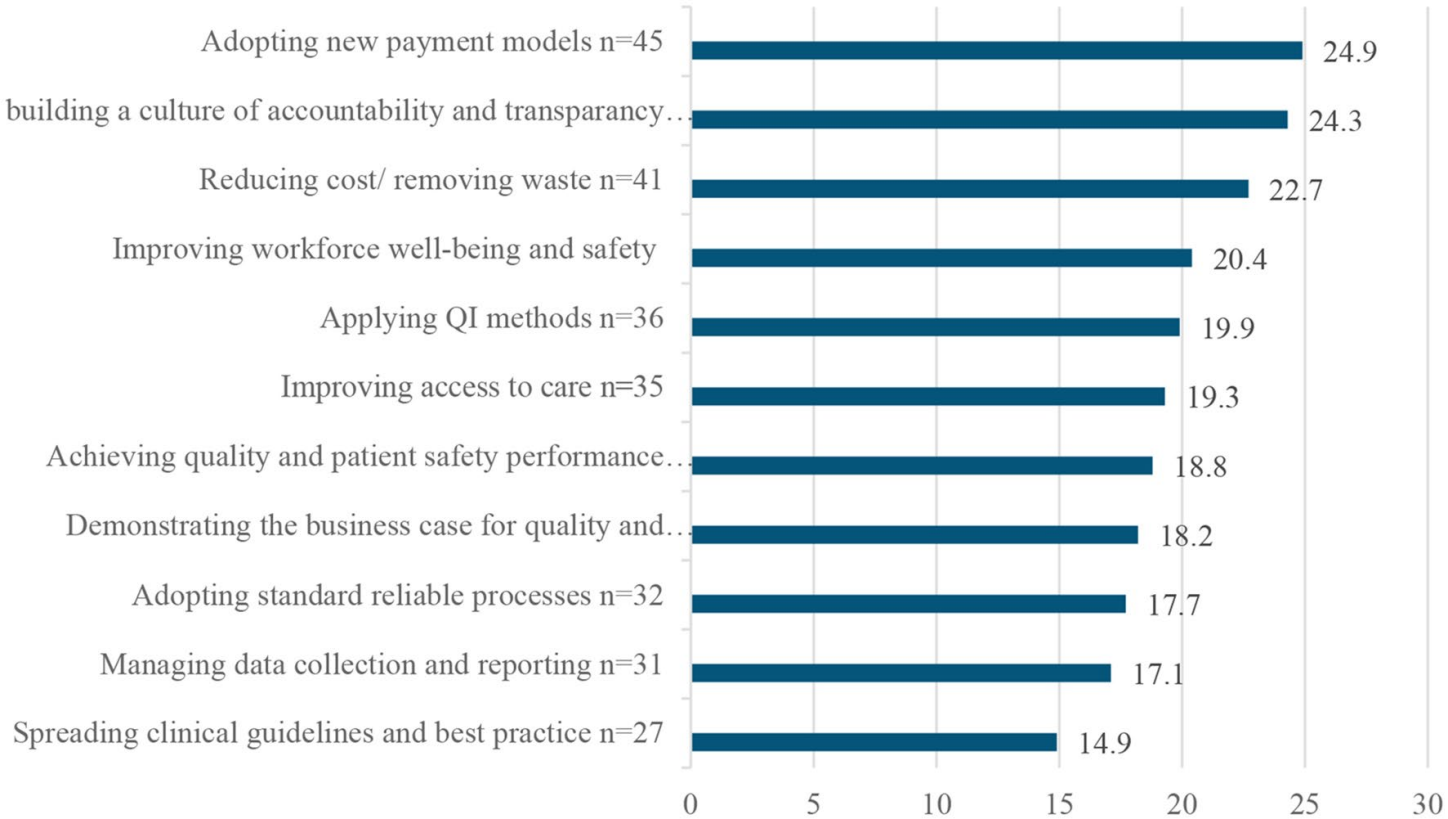
Implementation challenges in healthcare quality improvement as reported by clinicians

The top QI implementation challenges faced by healthcare professionals’ hospitals are detailed in (Fig. 3).

The results of investigating factors affecting healthcare professionals’ self-efficacy in conducting QI show a statistically significant difference in self-efficacy levels across age groups (p-value = 0.003). The two older age groups (34–49) and (50 and more), recorded a higher median score (Md = 4), than the younger [18–33] group (Md = 3). Years of experience also showed a significant difference (P-value = 0.038), and more than six years of experience recorded a higher Md score (Md = 4) than the less experienced groups. Healthcare professionals who had formal training in QI, professional workshops in QI, and QI organisational membership were more likely to report higher self-efficacy in conducting QI projects (p-value < 0.05) (Table 2).

**Table 2:** Factors affecting healthcare professionals’ self-efficacy in conducting quality improvement projects

Variable	Self-efficacy in conducting QI	
	Median (IQR)	*P* value
**Age in years**		0.003^b^
18–33	3 (3–4)	
34–49	4 (3–5)	
50 and more	4 (3–5)	
**Years of experience**		**0.038**
Less than 2 years	3 (3–4)	
2–6 Years	3 (3–4)	
More than 6 years	4 (3–5)	
**Formal QI training in hospital**		**0.010** ^**a**^
No	3 (3–4)	
Yes	4 (4–5)	
**Formal QI training outside hospital**		**0.025** ^**a**^
No	3 (3–4)	
Yes	5 (4–5)	
**Professional workshop or seminar in QI**		**0.010** ^**a**^
No	3 (3–4)	
Yes	4 (3–5)	
**QI organizational membership**		**0.035** ^**a**^
No	3 (3–4)	
Yes	4 (4–5)	

*P-value: significance level at 95% confidence interval*,* IQR: Interquartile range*, ^*a*^ *Mann-Whitney U test*, ^*b*^ *Kruskal-Wallis test*

## Discussion

This study addresses the facilitators, barriers, as well as opportunities to the engagement of healthcare professionals in quality improvement projects in Sudan. Data were collected from 1007 healthcare professionals (HCPs) representing health facilities in 18 states across Sudan. The majority of the participants were females, and most of them were physicians with less than two years of experience. Participants were stratified into three age groups; most of them were in the range of 18–33 years, with the mean age of 27 years.

Older age and increased years of clinical experience were found to be significantly associated with better experience in QI in this study, and it is consistent with a cross-sectional survey that investigated physicians’ interest and opportunities in QI work [19]. A possible explanation is that those with more years of experience have an increased tendency to get into decision-making and administrative roles, with more involvement in QI projects. On the contrary, junior clinicians primarily focus on providing patient care services during their working hours rather than engaging in QI projects.

It goes without saying that experience in QI correlates with its efficiency and success. In fact, experience is regarded as a key factor for successful QI projects [20, 21]. This aligns well with our study finding that prior experience with QI is the second most impactful factor in its success. One explanation is that healthcare providers with greater experience in QI exhibit higher self-efficacy, leading to more active and sustained participation in improvement efforts. According to a study assessing the Quality Improvement and Self-Efficacy Inventory (QISEI) among nurses, those with prior QI experience rated their competencies higher than those without such experience [22].

Interestingly, despite that the females constitute the vast majority in this study, the male HCPs show a higher experience with QI than females (24.1% against 15.1%). It’s a gender gap which is further emphasised and supported by other reports demonstrating that 70% of the health workforce are women, but they are still behind in QI experience when compared to men. This could be attributed in part to less female contribution in the higher administrative and decision-making roles in hospitals and public health systems [23, 24]. This gender disparity can be visualised as an outcome of social as well as organisational neglect. In many societies, lack of familial support, stereotyping, culture and work-life balance are among the main barriers for females to enrol in high responsibility positions in healthcare settings [25]. Even those who took the experience were challenged by a lack of institutional support, mentorship and experience [26]. If we are aiming to bridge this gap and optimise gender equity, we should enforce policies that advocate for women’s participation in leadership positions in healthcare systems.

Our findings show that professional location and employment setting have no significant effect on the QI participation. Yet these factors, among other contextual elements, should be considered when quality interventions are to be implemented. Previous studies showed that the context affects multiple aspects of QI projects and dictates their success [21, 27].

When barriers were investigated, the lack of organisational support was reported by 59.1% of the respondents as a barrier to quality improvement practice, followed by the inaccessibility to QI content, lack of time and lack of mentorship. Conversely, the lack of organisational support was the least frequently reported barrier in Blok’s study [28]. On the other hand, we have found that during a typical work week, nearly two-thirds of the participants spent most of their time in the clinical care of patients, and only a small portion of time is dedicated to QI activities and research. Only 18% of participants reported prior experience with quality improvement projects. As of the studies done by Shaikh and Blok, the lack of time was the dominant barrier [18, 28], and is found to be more considerable among specialities with higher time constraints, like surgery [29].

As part of their commitment to continuous professional development, hospitals grant dedicated time for engagement in QI opportunities to their employees, as stated by 45% of our participants. This is a lower percentage when compared to 77.8% reported by Sheikh et al. in their international study across 50 countries, but this variation is well justified by the fact that the respondents in Sheikh’s study were exclusively members and fellows of the International Society for Quality in Healthcare. So, they are QI activists and practitioners [18].

Formal QI training within and outside the specific organisation, mentorship, and networking opportunities in QI are not only methods utilised by the healthcare professionals as part of professional development, but they have also been regarded as factors of success of QI projects. Based on our results, successful QI projects are positively influenced by multiple factors, among which formal QI training and personal ability to identify problems are among the most frequent, aligning with a previous study [18]. Van Schie and colleagues, in their quality improvement initiative (QII), have concluded that QI success implies improved patient outcomes [30], which has been further supported by other studies [18, 31]. In fact, quality improvement is a two-way process; it ensures better quality of service as well as better professional performance of healthcare workers. Through QI activities, healthcare professionals acquire commitment, leadership and teamwork skills, knowledge and a problem-solving mindset [32].

Even though many medical schools have incorporated QI courses in their curriculum, the results show that further emphasis and training are needed. We recommend that the health authorities and organisations prioritise QI engagement and integrate it with the formal clinical training in attempts to enhance the quality of care and build the staff capacity to be able not only to participate, but also to lead quality improvement initiatives. It’s important to recognise that quality improvement needs substantial multidisciplinary efforts and involvement of leaders at different organisational levels to achieve the targeted quality of care. A well-organised audit and feedback system is essential to enforce the performance evaluation concepts among our health professionals [33].

It is debatable whether QI interventions are worthy investments due to their financial demands, yet they help prevent the economic burden and devastating humanitarian consequences of inaccessible high-quality care. Mortality and economic disadvantages are inequitably distributed, with a greater impact on the limited-resource countries [34]. In fact, low- and middle-income countries have their own contextual issues and difficulties regarding the adoption and implementation of quality improvement strategies. In such a busy environment where physicians and other health professionals are directing their full capacity to deliver the care, engagement in QI projects represents a true challenge [4].

In Sudan, as a limited-resource country, the adoption of and investment in quality improvement projects is still behind the global trends. The concept of QI is not fully developed in Sudan, and its application is way more challenging than in other limited-resource countries due to the ongoing crisis. Also, research in this subject is lacking, and no adequate database to reflect the situation. In October 2008, a group of healthcare leaders and quality experts agreed that the under-resourced countries need additional efforts to enroot the investment in quality interventions, including the formation of interest groups, the involvement of the stakeholders and addressing the barriers to quality improvement in healthcare [2].

### Strengths and limitations

Our current work is the first national study that tackles the engagement in QI projects from different healthcare professionals’ perspectives, laying the ground for further exploration and innovation of practical strategies to establish well-organised QI projects. There are some limitations to be mentioned here; since the engagement in QI activities shows a wide range of variation and different perspectives, further stratification with proportionate sampling based on the age, geographical distribution -the state- in this case, the professional location, the employment setting and the type of clinical training is needed to reflect the situation accurately. Additionally, some areas were underrepresented; for instance, we have only 4% of the responses from West Sudan, and this may be in part attributed to the political disadvantages and network instability over there. Furthermore, due to the sample constraints of HCPs with QI experience (*n* = 181), we used the non-parametric tests (Mann-Whitney U and Kruskal-Wallis) to compare self-efficacy across groups. While these tests detect the differences between groups, they do not provide effect sizes or adjust for confounders, limiting our ability to identify predictors of self-efficacy in QI. Future studies with larger samples could use regression-based methods to identify the predictors.

## Conclusion

A good clinician is not only one who prescribes the right medication, but also the one who knows how to identify system-related practice defects and manage them. QI engagement facilitators, barriers and opportunities are influenced and determined by a variety of factors that should be adjusted for when adopting and implementing QI initiatives. In low-resource settings, additional contextual barriers are expected and should be addressed during QI attempts. Successful QI projects largely contribute to the patients’ safety, well-being and improved outcomes. Also, it helps implement the guidelines and increases the effectiveness of practice while decreasing malpractice and medical errors. Further studies are needed to explore the future of QI in Sudan in the light of contextual factors in order to redesign and implement the globally adopted QI strategies.

## Supplementary Information

Below is the link to the electronic supplementary material.

Supplementary Material 1

## Data Availability

All data relevant to the study are de-identified and included in the article or uploaded as supplementary information.

## Abbreviations

QI: Quality Improvement
HCPs: Healthcare Professionals
WHO: World Health Organisation

## References

[1] Who. Everybody’s business: strengthening health systems to improve health outcomes: WHO’s framework for action. Production [Internet]. 2007 [cited 2025 Jun 11];1–56. Available from: http://www.who.int/healthsystems/strategy/everybodys_business.pdf

[2] Leatherman S, Ferris TG, Berwick D, Omaswa F, Cris P. The role of quality improvement in strengthening health systems in developing countries. Int J Qual Health Care [Internet]. 2010 Jun 12 [cited 2025 Jun 11];22(4):237–43. Available from: https://pubmed.ncbi.nlm.nih.gov/20543209/10.1093/intqhc/mzq028 20543209

[3] Iroz CB, Ramaswamy R, Bhutta ZA, Barach P. Quality improvement in public–private partnerships in low- and middle-income countries: a systematic review. BMC Health Services Research 2024 24:1 [Internet]. 2024 Mar 13 [cited 2025 Aug 9];24(1):1–16. Available from: https://bmchealthservres.biomedcentral.com/articles/10.1186/s12913-024-10802-w 10.1186/s12913-024-10802-w PMC10935959 38481226

[4] Garcia-Elorrio E, Schneider EC. Research on health-care quality improvement in low- and middle-income countries: is it a worthy investment? Int J Qual Health Care [Internet]. 2012 Dec [cited 2025 Jun 11];24(6):550–2. Available from: https://pubmed.ncbi.nlm.nih.gov/23139251/10.1093/intqhc/mzs067 23139251

[5] Massoumi S, Meshkati N, Placencia G, Sullivan KJ, Prestwich B. A Healthcare provider model to integrate human factors and patient safety in family home healthcare settings. Proceedings of the International Symposium on Human Factors and Ergonomics in Health Care [Internet]. 2015 Jun [cited 2025 Jun 11];4(1):182–7. Available from: 10.1177/2327857915041004. https://journals.sagepub.com/

[6] Foster T, Ogrinc G, Hamby L, Weeks WB. Improving the effectiveness of physician participation in local quality improvement efforts. Qual Manag Health Care [Internet]. 2002 [cited 2025 Jun 11];10(3):25–30. Available from: https://pubmed.ncbi.nlm.nih.gov/12512462/10.1097/00019514-200210030-00008 12512462

[7] Myers JS, Wong BM. Measuring outcomes in quality improvement education: success is in the eye of the beholder. BMJ Qual Saf [Internet]. 2019 May 1 [cited 2025 Jun 11];28(5):345–8. Available from: https://qualitysafety.bmj.com/content/28/5/345 10.1136/bmjqs-2018-008305 30886120

[8] Shojania KG, Grimshaw JM. Evidence-based quality improvement: the state of the science. Health Aff (Millwood) [Internet]. 2005 Jan [cited 2025 Jun 11];24(1):138–50. Available from: https://pubmed.ncbi.nlm.nih.gov/15647225/10.1377/hlthaff.24.1.138 15647225

[9] Davies H, Powell A, Rushmer R. Why don’t clinicians engage with quality improvement? J Health Serv Res Policy [Internet]. 2007 Jul 1 [cited 2025 Jun 11];12(3):129–30. Available from: https://journals.sagepub.com/doi/10.1258/135581907781543139 10.1258/135581907781543139 17716412

[10] Elizalde J, Lumibao J, Lizarondo L. Barriers and facilitators to health professionals’ engagement in quality improvement initiatives: a mixed-methods systematic review. Int J Qual Health Care [Internet]. 2024 Apr 1 [cited 2025 Jun 11];36(2). Available from: https://pubmed.ncbi.nlm.nih.gov/38727534/10.1093/intqhc/mzae041 38727534

[11] War. in Sudan has displaced over 14 million, or about 30% of the population, UN says | AP News [Internet]. [cited 2025 Jun 11]. Available from: https://apnews.com/article/sudan-war-refugees-displaced-un-8672055b2a23b019662e8005a8c54e77

[12] Inside Sudan’s forgotten war. 150,000 dead, 11 million displaced [Internet]. [cited 2025 Jun 11]. Available from: https://www.thetimes.com/world/africa/article/sudan-forgotten-war-khartoum-omdurman-z5zn33nmh

[13] Sudan | OCHA [Internet]. [cited 2025 Jun 11]. Available from: https://www.unocha.org/sudan

[14] ‘Deeply inspiring and humbling’. how neighbourhoods in Sudan are coming together to fill gaps left by foreign aid | Global development | The Guardian [Internet]. [cited 2025 Jun 11]. Available from: https://www.theguardian.com/global-development/2024/dec/31/neighbourhoods-sudan-gaps-foreign-aid-community-kitchen-emergency-response-rooms

[15] von Elm E, Altman DG, Egger M, Pocock SJ, Gøtzsche PC, Vandenbroucke JP. Strengthening the reporting of observational studies in epidemiology (STROBE) statement: guidelines for reporting observational studies. BMJ: British Medical Journal [Internet]. 2007 Oct 20 [cited 2024 Dec 16];335(7624):806. Available from: https://pmc.ncbi.nlm.nih.gov/articles/PMC2034723/10.1136/bmj.39335.541782.AD PMC2034723 17947786

[16] Bornstein MH, Jager J, Putnick DL. Sampling in developmental science: situations, shortcomings, solutions, and standards. Dev Rev. 2013;33(4):357–70. 25580049 10.1016/j.dr.2013.08.003 PMC4286359

[17] Wu Suen LJ, Huang HM, Lee HH. A comparison of convenience sampling and purposive sampling. Hu Li Za Zhi [Internet]. 2014 Jun 1 [cited 2025 Aug 9];61(3):105–11. Available from: https://pubmed.ncbi.nlm.nih.gov/24899564/10.6224/JN.61.3.105 24899564

[18] Shaikh U, Lachman P, Padovani AJ, McCarthy SE. The care and keeping of clinicians in quality improvement. Int J Qual Health Care [Internet]. 2020 Aug 1 [cited 2025 Jun 11];32(7):480–5. Available from: https://pubmed.ncbi.nlm.nih.gov/32613236/10.1093/intqhc/mzaa071 32613236

[19] Deilkås ET, Rosta J, Baathe F, Søfteland E, Lexberg ÅS, Røise O et al. Physician participation in quality improvement work- interest and opportunity: a cross-sectional survey. BMC Primary Care [Internet]. 2022 Dec 1 [cited 2025 Jun 11];23(1):1–9. Available from: https://bmcprimcare.biomedcentral.com/articles/10.1186/s12875-022-01878-6 10.1186/s12875-022-01878-6 PMC9594954 36284296

[20] Kaplan HC, Brady PW, Dritz MC, Hooper DK, Linam WM, Froehle CM et al. The influence of context on quality improvement success in health care: a systematic review of the literature. Milbank Q [Internet]. 2010 Dec [cited 2025 Jun 11];88(4):500–59. Available from: https://pubmed.ncbi.nlm.nih.gov/21166868/10.1111/j.1468-0009.2010.00611.x PMC3037175 21166868

[21] Kaplan HC, Provost LP, Froehle CM, Margolis PA. The model for understanding success in quality (MUSIQ): building a theory of context in healthcare quality improvement. BMJ Qual Saf [Internet]. 2012 Jan [cited 2025 Jun 11];21(1):13–20. Available from: https://pubmed.ncbi.nlm.nih.gov/21835762/10.1136/bmjqs-2011-000010 21835762

[22] Baernholdt M, Jones TL, Anusiewicz CV, Campbell CM, Montgomery A, Patrician PA. Development and testing of the quality improvement self-efficacy inventory. West J Nurs Res [Internet]. 2022 Feb 1 [cited 2025 Jun 11];44(2):159–68. Available from: https://pubmed.ncbi.nlm.nih.gov/33745388/10.1177/0193945921994158 PMC8450303 33745388

[23] Betron M, Bourgeault I, Manzoor M, Paulino E, Steege R, Thompson K et al. Time for gender-transformative change in the health workforce. Lancet [Internet]. 2019 Feb 9 [cited 2025 Jun 11];393(10171):e25–6. Available from: https://pubmed.ncbi.nlm.nih.gov/30739706/10.1016/S0140-6736(19)30208-9 30739706

[24] Pérez-Sánchez S, Maduenõ SE, Montaner J. Gender gap in the leadership of health institutions: The influence of hospital-level factors. Health equity [Internet]. 2021 Aug 1 [cited 2025 Jun 11];5(1):521. Available from: https://pmc.ncbi.nlm.nih.gov/articles/PMC8409238/10.1089/heq.2021.0013 PMC8409238 34476325

[25] Kalaitzi S, Czabanowska K, Azzopardi-Muscat N, Cuschieri L, Petelos E, Papadakaki M et al. Women, healthcare leadership and societal culture: a qualitative study. J Healthc Leadersh [Internet]. 2019 [cited 2025 Jun 11];11:43. Available from: https://pmc.ncbi.nlm.nih.gov/articles/PMC6469472/10.2147/JHL.S194733 PMC6469472 31043802

[26] Shillcutt SK, Parangi S, DIekman S, Ghalib R, Schoenthaler R, Girgis LM et al. Survey of women physicians’ experience with elected leadership positions. Health equity [Internet]. 2019 [cited 2025 Jun 11];3(1):162. Available from: https://pmc.ncbi.nlm.nih.gov/articles/PMC6608691/10.1089/heq.2018.0101 PMC6608691 31289775

[27] Silver SA, McQuillan R, Harel Z, Weizman AV, Thomas A, Nesrallah G et al. How to sustain change and support continuous quality improvement. Clin J Am Soc Nephrol [Internet]. 2016 [cited 2025 Jun 11];11(5):916–24. Available from: https://pubmed.ncbi.nlm.nih.gov/27016498/10.2215/CJN.11501015 PMC4858491 27016498

[28] Blok AC, Alexander CC, Tschannen D, Milner KA. Quality improvement engagement: barriers and facilitators. Nurs Manage [Internet]. 2022 Mar 1 [cited 2025 Jun 11];53(3):16–24. Available from: https://pubmed.ncbi.nlm.nih.gov/35225833/10.1097/01.NUMA.0000821708.46746.6f 35225833

[29] Wolfstadt JI, Cohen-Rosenblum A. ‘You can’t do quality between surgical cases and tea time’: barriers to surgeon engagement in quality improvement. BMJ Qual Saf [Internet]. 2023 Jan 1 [cited 2025 Jun 11];32(1):10–2. Available from: https://qualitysafety.bmj.com/content/32/1/10 10.1136/bmjqs-2022-015083 36549699

[30] Correction. Effectiveness of a multifaceted quality improvement intervention to improve patient outcomes after total hip and knee arthroplasty: a registry nested cluster randomised controlled trial. BMJ Qual Saf [Internet]. 2022 Sep 5 [cited 2025 Jun 11];31(11):E1. Available from: https://pubmed.ncbi.nlm.nih.gov/38467425/10.1136/bmjqs-2021-014472corr1 38467425

[31] Pronovost P, Needham D, Berenholtz S, Sinopoli D, Chu H, Cosgrove S et al. An intervention to decrease catheter-related bloodstream infections in the ICU. N Engl J Med [Internet]. 2006 Dec 28 [cited 2025 Jun 11];355(26):2725–32. Available from: https://pubmed.ncbi.nlm.nih.gov/17192537/10.1056/NEJMoa061115 17192537

[32] Zamboni K, Baker U, Tyagi M, Schellenberg J, Hill Z, Hanson C. How and under what circumstances do quality improvement collaboratives lead to better outcomes? A systematic review. Implement Sci [Internet]. 2020 May 4 [cited 2025 Jun 11];15(1). Available from: https://pubmed.ncbi.nlm.nih.gov/32366269/10.1186/s13012-020-0978-z PMC7199331 32366269

[33] Kirchner JAE, Parker LE, Bonner LM, Fickel JJ, Yano EM, Ritchie MJ. Roles of managers, frontline staff and local champions, in implementing quality improvement: stakeholders’ perspectives. J Eval Clin Pract [Internet]. 2012 Feb [cited 2025 Jun 11];18(1):63–9. Available from: https://pubmed.ncbi.nlm.nih.gov/20738467/10.1111/j.1365-2753.2010.01518.x 20738467

[34] Alkire BC, Peters AW, Shrime MG, Meara JG. The economic consequences of mortality amenable to high-quality health care in low- and middle-income countries. Health Aff (Millwood) [Internet]. 2018 Jun 1 [cited 2025 Jun 11];37(6):988–96. Available from: https://pubmed.ncbi.nlm.nih.gov/29863936/10.1377/hlthaff.2017.1233 29863936

