# Supplementary material for: Improving healthcare quality in Sudan: situation and factors influencing healthcare professionals’ engagement
Source: BMC Health Serv Res. 2025 Sep 30;25:1237. doi: 10.1186/s12913-025-13481-3 (PMC12482710; doi:10.1186/s12913-025-13481-3)
Supplement: Supplementary file 1 — Supplementary Material 1 [file 12913_2025_13481_MOESM1_ESM.docx]

| **Variable** | **Self-efficacy** | |
| --- | --- | --- |
|  | **Median (IQR)** | **P value** |
| **Gender** |  | 0.337^a^ |
| Male | 4 (3-4) |  |
| Female | 3 (3-4) |  |
| **Age in Years** |  | **0.003^b^** |
| 18-33 | 3 (3-4) |  |
| 34-49 | 4 (3-5) |  |
| 50 and more | 4 (3-5) |  |
| **Did you receive a course in QI before graduation?** |  | 0.510^a^ |
| No | 3 (3-4) |  |
| Yes | 3 (3-4) |  |
| **The hospital provides dedicated time for clinicians to participate in QI projects** |  | 0.152^a^ |
| No | 3 (3-4) |  |
| Yes | 4 (3-5) |  |
| **The hospital rewards participation clinicians' participation in QI projects** |  | 0.921^a^ |
| Yes | 3 (3-4) |  |
| No | 3 (3-4) |  |
| **Professional location** |  | 0.445^b^ |
| Urban | 3 (3-4) |  |
| Rural | 4 (3-5) |  |
| Suburb | 3 (2-5) |  |
| **Clinical training** |  | 0.892^b^ |
| Physician | 3 (3-4) |  |
| Nurse | 3 (3-4) |  |
| Pharmacist | 4 (3-4) |  |
| Lab scientist | 4 (3-5) |  |
| Physical therapist | 3 (2-4) |  |
| **Employment Setting** |  | 0.054^b^ |
| State government hospital | 3 (3-4) |  |
| Private hospital | 4 (3-5) |  |
| University health center | 3 (3-4) |  |
| Community health center | 4 (4-5) |  |
| **Region** |  | 0.476^b^ |
| Khartoum | 4 (3-4) |  |
| North Sudan | 3 (3-4) |  |
| Central Sudan | 3 (2-5) |  |
| West Sudan | 5 (3-5) |  |
| East Sudan | 3 (3-4) |  |
| **Years of Experience** |  | **0.038^b^** |
| Less than 2 years | 3 (3-4) |  |
| 2-6 Years | 3 (3-4) |  |
| More than 6 years | 4 (3-5) |  |
| **Formal QI training in Hospital** |  | **0.010^a^** |
| No | 3 (3-4) |  |
| Yes | 4 (4-5) |  |
| **Formal QI training outside hospital** |  | **0.025^a^** |
| No | 3 (3-4) |  |
| Yes | 5 (4-5) |  |
| **Professional workshop or seminar in QI** |  | **0.010^a^** |
| No | 3 (3-4) |  |
| Yes | 4 (3-5) |  |
| **QI conference** |  | 0.153^a^ |
| No | 3 (3-4) |  |
| Yes | 4 (3-5) |  |
| **Online course in QI** |  | 0.205^a^ |
| No | 3 (3-4) |  |
| Yes | 4 (3-4) |  |
| **QI organizational membership** |  | **0.035^a^** |
| No | 3 (3-4) |  |
| Yes | 4 (4-5) |  |
| **Mentorship in QI** |  | 0.762^a^ |
| No | 3 (3-4) |  |
| Yes | 4 (3-4) |  |
| *P-value: significance level at 95% confidence interval, IQR: Interquartile range, ^a^ Mann-Whitney U test, ^b^ Kruskal-Wallis test* | | |
